# Incident infection following acute kidney injury with recovery to baseline creatinine: A propensity score matched analysis

**DOI:** 10.1371/journal.pone.0217935

**Published:** 2019-06-24

**Authors:** Benjamin R. Griffin, Zhiying You, John Holmen, Megan SooHoo, Katja M. Gist, James F. Colbert, Michel Chonchol, Sarah Faubel, Anna Jovanovich

**Affiliations:** 1 Division of Renal Diseases and Hypertension, University of Colorado Denver Anschutz Medical Campus, Aurora, CO, United States of America; 2 Intermountain Healthcare System, Salt Lake City, UT, United States of America; 3 Department of Pediatrics, Children’s Hospital Colorado, Aurora, CO, United States of America; 4 Division of Infectious Diseases, University of Colorado Denver Anschutz Medical Campus, Aurora, CO, United States of America; 5 Renal Section, VA Eastern Colorado Health Care System, Denver, CO, United States of America; University of Wisconsin, UNITED STATES

## Abstract

**Background:**

Severe acute kidney injury (AKI) is associated with subsequent infection. Whether AKI followed by a return to baseline creatinine is associated with incident infection is unknown.

**Objective:**

We hypothesized that risk of both short and long term infection would be higher among patients with AKI and return to baseline creatinine than in propensity score matched peers without AKI in the year following a non-infectious hospital admission.

**Design:**

Retrospective, propensity score matched cohort study.

**Participants:**

We identified 494 patients who were hospitalized between January 1, 1999 and December 31, 2009 and had AKI followed by return to baseline creatinine. These were propensity score matched to controls without AKI.

**Main Measures:**

The predictor variable was AKI defined by *International Classification of Diseases*, *Ninth Revision* (*ICD-9*) codes and by the Kidney Disease Improving Global Outcomes definition, with return to baseline creatinine defined as a decrease in serum creatinine level to within 10% of the baseline value within 7 days of hospital discharge. The outcome variable was incident infection defined by ICD-9 code within 1 year of hospital discharge.

**Results:**

AKI followed by return to baseline creatinine was associated with a 4.5-fold increased odds ratio for infection (odds ratio 4.53 [95% CI, 2.43–8.45]; p<0.0001) within 30 days following discharge. The association between AKI and subsequent infection remained significant at 31–60 days and 91 to 365 days but not during 61–90 days following discharge.

**Conclusion:**

Among patients from an integrated health care delivery system, non-infectious AKI followed by return to baseline creatinine was associated with an increased odds ratio for infection in the year following discharge.

## Introduction

Acute kidney injury (AKI) is a common and widely recognized cause of morbidity and mortality in hospitalized patients. The incidence of AKI has been increasing over the past decade and is now observed in up to 20% of all inpatient admissions [1–3], which amounts to approximately 17 million cases annually in the United States[4]. Furthermore, AKI has been shown to independently increase in-hospital morbidity and mortality[3, 5–7], which adds an estimated $10 billion in additional health care expenditures annually[4].

In the past, AKI was considered a self-limited disease resulting from a temporary loss of renal clearance with negative impacts mediated through fluid and electrolyte imbalances[8]. Today AKI is increasingly recognized as a systemic disease that affects multiple organs including the lungs, heart, and brain[9]. A growing body of literature shows that AKI is associated with remote organ injury in the acute setting as well as in the long term. Indeed, AKI is associated with long term morbidity and mortality[10–12] including increased incidence of chronic kidney disease (CKD)[13–15] and cardiovascular events[16–18], and it may also be linked with higher rates of cancer[19], active tuberculosis[20], bone fracture[21], and stroke[22].

Several studies clearly demonstrate that AKI is associated with increased short-term rates of infection[7, 23–27]. Acute kidney injury may also be associated with increased long-term risk of infection[20, 27]. Studies to date have focused on the increased rates of infection in patients with AKI who required renal replacement therapy (RRT), a group at increased risk in part due to the need for central lines and dialysis catheters. These patients are also at high risk for subsequent development of CKD, which itself is associated with increased infection risk. Even in studies looking at outcomes in patients with renal recovery, the definition used was cessation of hemodialysis, not a full return to pre-hospitalization baseline creatinine. The risk of long-term infection has not been evaluated in patients with non-RRT requiring AKI with a return to baseline creatinine at the time of discharge.

To better assess the association of AKI and both short and long-term incident infection, we compared patients with return to baseline creatinine after an episode of AKI to patients without AKI within an integrated health care delivery system. We hypothesized that the risk of infection in the year following a hospital admission complicated by AKI with return to baseline creatinine would be higher than propensity-score matched controls without AKI.

## Materials and methods

### Data source

We performed a retrospective cohort study using the Intermountain Healthcare Enterprise Data Warehouse, which incorporates comprehensive health and administrative data. Intermountain Healthcare is a not-for-profit system with more than 180 outpatient clinics, 22 hospitals, and averages more than 130,000 admissions annually. Its facilities range from major adult tertiary-level care centers to small clinics and hospitals that are the only source of care in rural communities[28]. Details regarding the data source, cohort definition, and statistical methods have been previously published[15].

### Cohort definition

**Fig 1:**
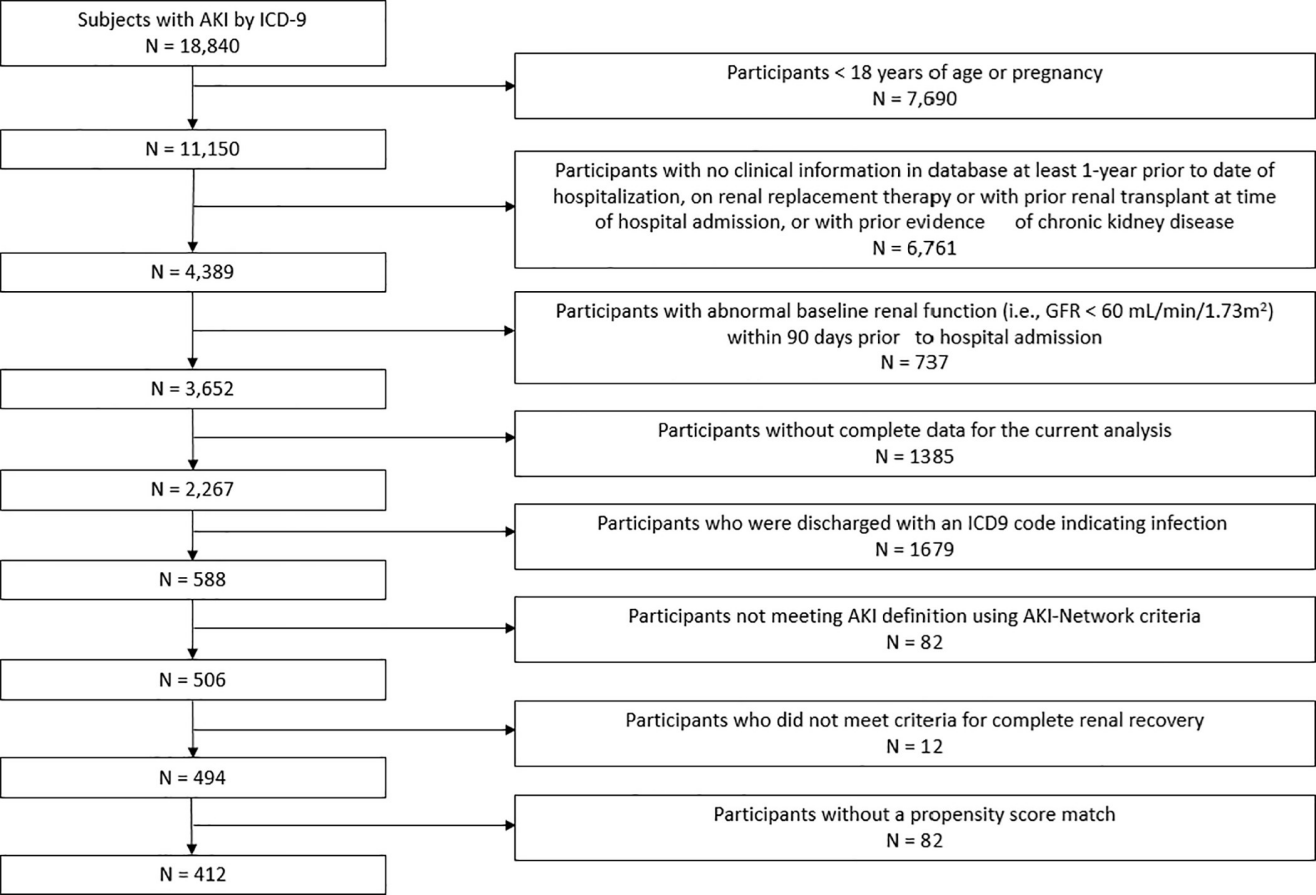
Cohort definition.

The study sample included all adult patients who had at least one hospitalization between January 1, 1999 and December 31, 2009. The Institutional Review Board at Intermountain Healthcare System and University of Colorado approved the project with a waiver of informed consent. All participants (AKI and propensity-score matched controls) were required to have administrative and clinical data, including at least one creatinine value, in the Intermountain Healthcare system at least 90 days prior to the index hospital admission. We defined the first AKI hospitalization in this period as the index admission, with follow-up beginning at the time of discharge from the inpatient service. We excluded patients who were younger than 18 years of age or who were pregnant. We also excluded patients who had an inpatient procedure code for acute hemodialysis, a prior diagnosis of AKI, a diagnosis of end-stage renal disease (ESRD), or an estimated glomerular filtration rate (GFR) <60 mL/min/1.73 m^2^ by the CKD-EPI (CKD Epidemiology Collaboration) prediction equation[29]. All serum creatinine measurements in the Intermountain Healthcare system used a modified kinetic Jaffe method[30]. Finally, all participants (AKI and controls) with an index hospitalization discharge ICD-9 code that indicated infection were also excluded. Fig 1 shows the number of participants with AKI excluded at each step and the final study population.

### Definitions

Cases of AKI were identified if they had both an *International Classification of Diseases*, *Ninth Revision* (ICD-9) code for AKI (584.0, 584.5, 584.9, and 580.9) on an inpatient claim[31] and the Kidney Disease Improving Global Outcomes (KDIGO) definition for AKI based on inpatient serum creatinine. AKI participants were identified within the hospitalization by comparing the highest serum creatinine value during the hospitalization period (between admission and discharge dates) with the lowest serum creatinine concentration available 90 days before the index admission (baseline creatinine level). When the hospitalization value to the baseline creatinine value exceeded the baseline value by ≥ 0.3 mg/dL and the patient had an ICD-9 code indicating AKI, the participant was classified as an AKI case. AKI events were categorized further by KDIGO stage (I, II, III). The approach of identifying AKI participants using both ICD-9 codes and the KDIGO definition allowed us to confirm the presence of AKI based on objective criteria, and it also confirmed that a physician noted the AKI during the hospitalization. This approach was superior to using creatinine values alone because it reduced potential bias related to disease recognition. Patients who did not meet both criteria were not included in the AKI group.

Return to baseline creatinine after AKI was defined as return of serum creatinine to within 10% of the baseline value within 7 days of the discharge date.

### Outcomes

The primary outcome was incident infection during four different time periods following discharge: 1–30, 31–60, 61–90, and 91–365 days after the discharge from the index hospitalization. Incident infection was defined by ICD-9 codes (S1 Table) and categorized into 5 major groups: gastrointestinal and genitourinary infections; septicemia and bacteremia; skin, bone and joint infections; endocarditis; and pneumonia and other respiratory tract infections (S2 Table). An infectious episode was defined as one or more infections originating within a 15-day period. To address potential confounding due to partially treated infection, infection leading to infection, or an infectious episode lasting greater than 15 days, we performed survival analysis to determine time to first infection among AKI cases compared to controls.

### Statistical analysis

Descriptive statistics were used to summarize baseline characteristics of AKI and control participants. Baseline characteristics of AKI and control participants were compared using analysis of variance for continuous variables and chi-squared statistics for categorical variables. Follow-up started at the time of discharge for the index hospitalization and concluded 1 year later. All follow-up was completed by December 31, 2010.

We used logistic regression to calculate an estimate of the conditional probability of being in the AKI or control group given the selected variables (model c-statistic = 0.83), and the logit of the probability was used as the propensity score in matching. The variables used for the development of the propensity score included: age, sex, race, each individual component of the Charlson Comorbidity index[32], systolic blood pressure >140 mmHg, prior inpatient visits, admission season, baseline serum creatinine, and index hospitalization length of stay (LOS) in days. Using nearest neighbor matching, participants with AKI were 1:1 matched with control participants with a caliper distance of 0.05 standard deviation of the score without replacement. We the used the generalized estimating equation (GEE) procedure to estimate the odds of incident infection among those with AKI followed by return to baseline creatinine compared to controls. To determine time to first infection, a gamma frailty model was fitted by using SAS PROC PHREG with a Bayesian analysis. We considered a 2-sided p-value of 0.05 to be statistically significant. All statistical analyses were performed with SAS software version 9.4 (SAS Institute, Cary, NC).

## Results

### Baseline characteristics

There were 494 patients with AKI followed by return to baseline creatinine (cases) who met inclusion criteria during the study period. Of the 494, 412 cases were able to match to a control and were included in the analysis. Baseline characteristics of the AKI and control groups are listed in Table 1. The total study population had a mean age of 61 ± 18 years and was principally white (93%) with a slight male predominance (57%). The matched propensity score analysis did not show residual evidence of confounding in any of the matched categories (all *P*-values ≥0.05 after matching). (Table 1).

**Table 1:** Baseline characteristics of study participants after propensity-score matching.

	Case (AKI) N = 412	Control N = 412	P-value before matching*	P-value after matching
Age, years	61 ± 17	62 ± 18	<0.0001	0.06
Male	240 (58)	227 (55)	<0.0001	0.1
Caucasian	380 (92)	381 (93)	0.3	0.8
Myocardial infarction	71 (17)	77 (19)	<0.0001	0.6
Congestive heart failure	128 (31)	131 (29)	<0.0001	0.5
Peripheral vascular disease	73 (18)	83 (20)	<0.0001	0.9
Cerebrovascular disease	72 (18)	71 (17)	<0.0001	0.9
Dementia	10 (2)	10 (2)	0.5	0.7
Chronic pulmonary disease	212 (48)	215 (49)	<0.0001	0.8
Connective tissue disorder	28 (7)	35 (9)	0.1	0.4
Peptic ulcer disease	52 (13)	42 (10)	<0.0001	0.3
Diabetes, no complications	38 (9)	40 (10)	<0.0001	0.8
Diabetes, complications	142 (35)	152 (37)	<0.0001	0.4
Liver disease, mild	99 (24)	90 (22)	<0.0001	*0*.*6*
Liver disease, moderate to severe	19 (5)	17 (4)	<0.0001	0.7
Cancer, not metastatic	96 (23)	103 (25)	0.5	0.6
Cancer, metastatic	38 (9)	42 (10)	0.2	0.6
HIV/AIDS	0 (0.0)	0 (0)	0.2	NA
Paralysis	12 (3)	13 (3)	0.2	0.8
Systolic blood pressure >140 mmHg	302 (73)	321 (78)	<0.0001	0.06
Admission season			0.9	0.5
Winter	94 (23)	87 (21)		
Spring	109 (27)	108 (26)		
Summer	89 (24)	96 (23)		
Fall	112 (27)	120 (29)		
Prior inpatient visits	3.1 ± 3.2	3.2 ± 5.1	<0.0001	0.9
Index hospitalization LOS (days)	5.2 ± 3.9	4.9 ± 5.0	<0.0001	0.3
Baseline serum creatinine (mg/dL)	0.93 ± 0.17	0.94 ± 0.18	<0.0001	0.7

Data are presented as N (%) and mean ± SD.

* Comparing 2,180 controls and 494 cases prior to matching

Among cases baseline eGFR was 83 ± 18 mL/min/1.73m^2^ and post-hospitalization eGFR was 81 ± 19 mL/min/1.73m^2^, while in controls baseline and post- hospitalization eGFR was 81 ± 19 mL/min/1.73m^2^ and 86 ± 19 mL/min/1.73m^2^, respectively. Table 2 shows median baseline minimum serum creatinine, maximum encounter creatinine, and recovery creatinine for the control group and for the cases by KDIGO stage. Among the AKI cases, 165 (40%) had KDIGO stage I, 126 (31%) had KDIGO stage II, and 121 (29%) had KDIGO stage III. There were statistically significant differences in baseline creatinine among the controls and those who developed each stage of AKI, although these small differences are unlikely to be clinically significant. While all patients included in the study recovered to within 10% of their baseline creatinine value, the median recovery values were significantly higher in those with KDIGO stage I and II compared to controls; however, these differences are also unlikely to be clincally significant. At one year 33 cases and 5 controls had new CKD defined by eGFR <60 mL/min/1.73m^2^ on two occasions ≥90 days apart.

**Table 2:** Serum creatinine values during study period and AKI by KDIGO stage.

	Control	KDIGO Stage 1	p-value	KDIGO Stage 2	p-value	KDIGO Stage 3	p-value
	N = 412	N = 165	(stage 1 vs. control)	N = 126	(stage 2 vs. control)	N = 121	(Stage 3 vs. control)
Minimum Baseline SCr (mg/dL)	0.90 [0.80–1.10]	1.00 [0.85–1.10]	0.004	0.81 [0.70–1.00]	0.0004	0.80 [0.70–0.93]	<0.0001
Maximum Encoutner Scr (mg/dL)	0.90 [0.80–1.01]	1.50 [1.37–1.70]	<0.0001	2.00 [1.70–2.40]	<0.0001	4.50 [2.80–4.70]	<0.0001
Minimum Recovery SCr (mg/dL)	0.82 [0.70–1.00]*	0.90 [0.80–1.10]	<0.0001	0.87 [0.70–1.00]	0.05	0.90 [0.70–1.00]	0.1

Values given as median [IQR]. The p-value indicates statistical comparison of each KDIGO stage with the control group.

Abbreviations: KDIGO; Kidney Disease Improving Global Outcomes; Scr, serum creatinine

* N = 282

### Association between AKI with return to baseline creatinine and incident infection

**Fig 2:**
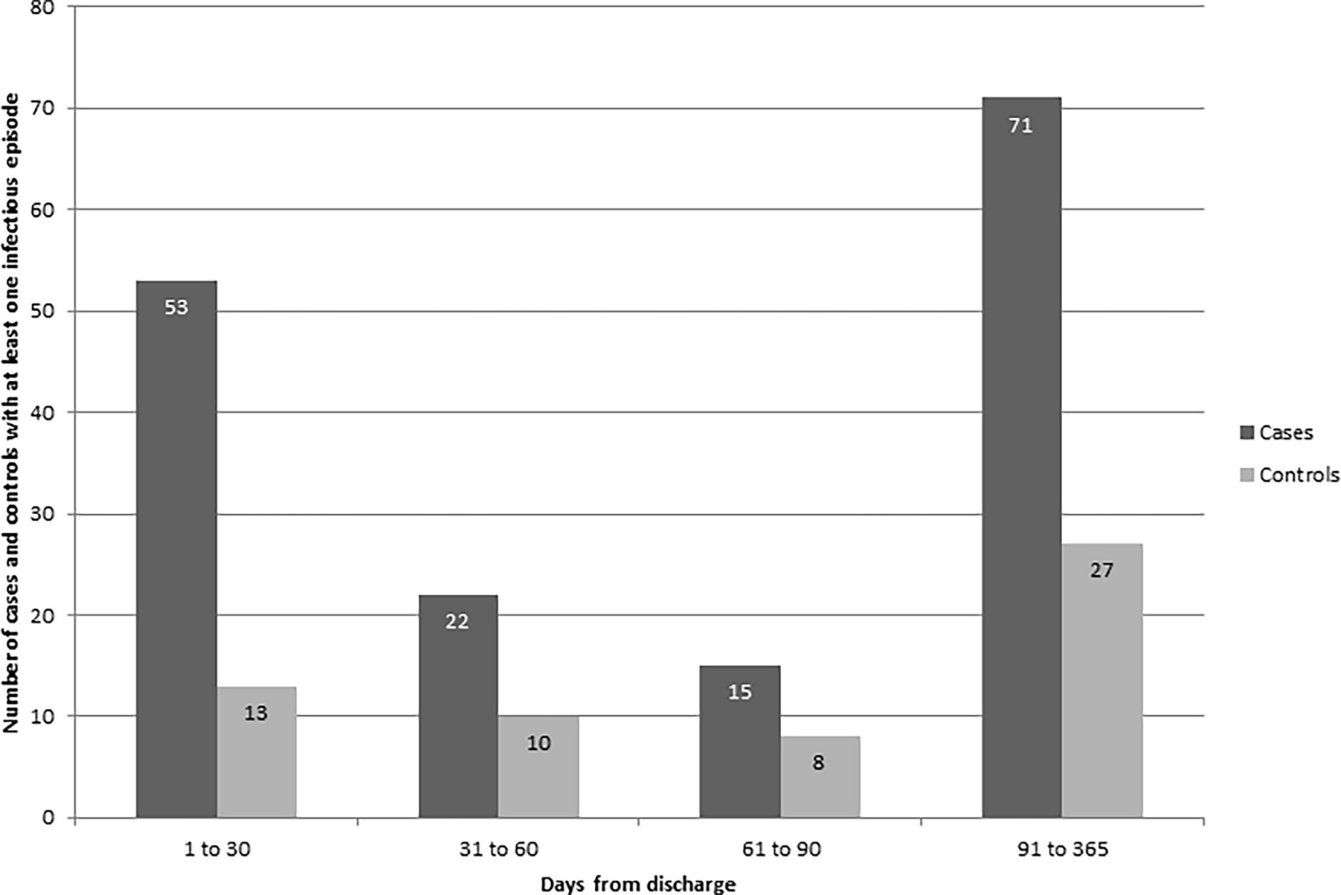
Number of cases and controls with at least one infectious episode at 1 to 30 days, 31 to 60 days, 61 to 90 days, and 91 to 365 days following discharge from the index hospitalization.

Within one year following discharge, AKI patients had more discreet infections (defined by ICD-9 code in a given 15-day period) than controls (*P* < 0.001). The major types of infections are listed in Table 3. For each time period following discharge, 1–30 days, 31–60 days, 60–90 days, and 91–365 days, more AKI cases had infectious episodes (defined as one or more infections originating within a 15-day period) compared to controls (Fig 2). Incident infection decreased for cases and controls at each 30-day comparison time period in the first 90 days. Incident infection increased among both cases and controls from 91–365 days likely due to the longer observation period.

**Table 3:** Number of discrete infections among cases and controls by major infection category in the year after discharge.

	Case (AKI) N = 412	Control N = 412
Endocarditis	3	0
Gastrointestinal and Genitourinary	91	30
Septicemia and Bacteremia	26	4
Pneumonia and Lung Infection	84	20
Skin, Bone, and Joint Infections	40	14
Total discrete infections	244	68

During the first 30 days post discharge, cases had a 4.5-fold increased odds ratio for incident infection compared to propensity-score matched controls (odds ratio [OR] 4.53, 95% CI 2.43–8.45, p <0.0001). Similarly, during the second 30-day period (days 31–60) following discharge, cases had a significantly greater odds ratio for incident infection (OR 3.46, 95% CI 1.02–11.71, p = 0.046). However, during the third 30-day period (days 61–90) there was no significant difference in incident infection between cases and controls (OR 2.75, 95% CI 0.75–10.10, p = 0.1). During the final observation period (91–365 days) following discharge, a greater odds ratio for incident infection was again observed among cases compared to controls (OR 9.75, 95% CI 2.98–31.49, p = 0.0002). Table 4 depicts ORs at each comparison time period. There was no significant association between the KDIGO stage during the index hospitalization and incident infection.

**Table 4:** Odds of incident infection among AKI cases compared to propensity score matched controls.

Time after index admission	Odds Ratio (95% Confidence Interval)	p-value
1–30 days	4.53 (2.43–8.45)	<0.0001
31–60 days	3.46 (1.02–11.71)	0.046
61–90 days	2.75 (0.75–10.10)	0.1
91–365 days	9.75 (2.98–31.485)	0.0002

**Fig 3:**
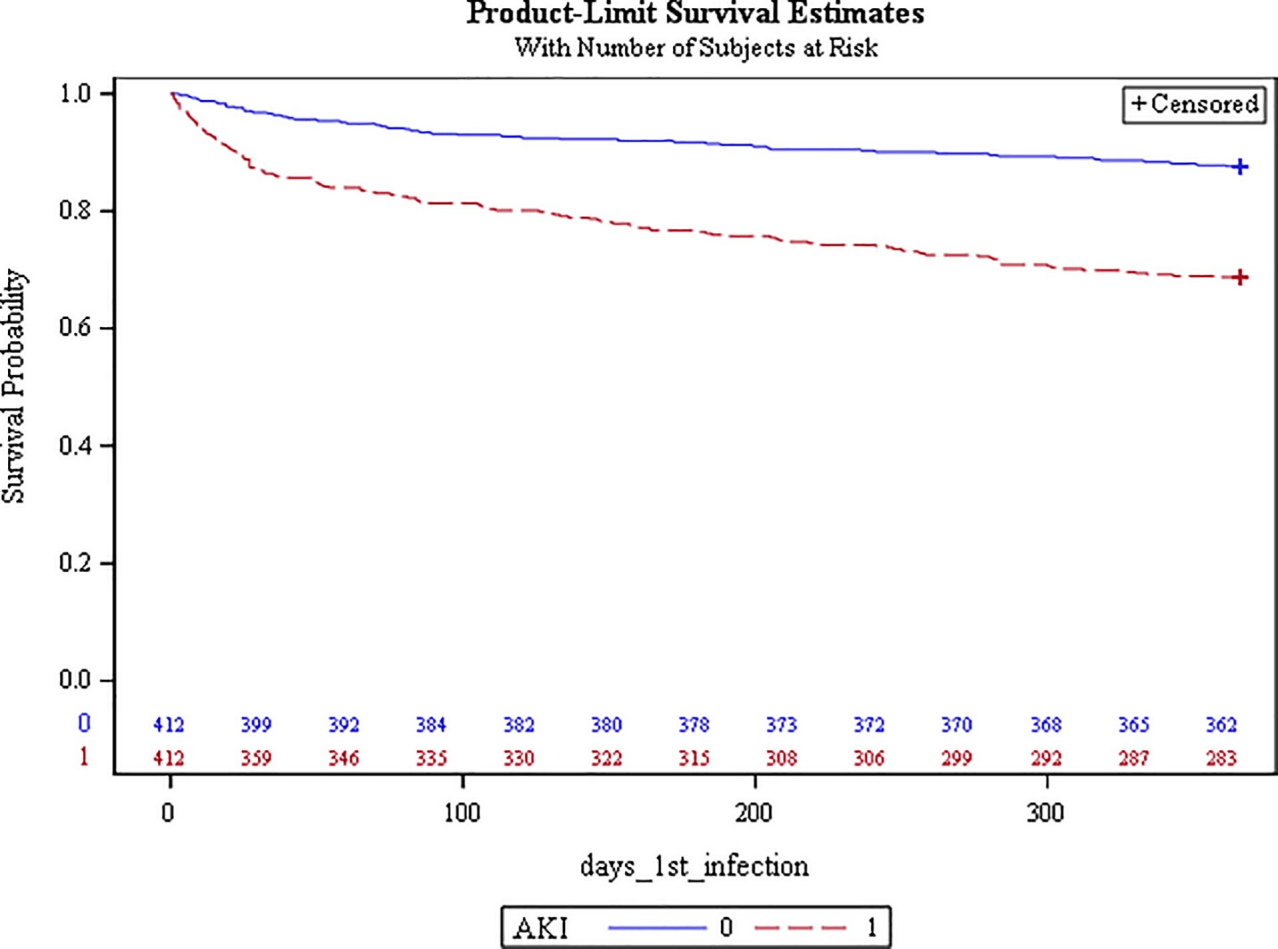
Survival curve for time to first infection among AKI cases (red) and controls (blue).

In survival analysis, which censored patients after the first infectious episode and thereby reduced confounding by recurrent or prolonged infection, patients with AKI followed by return to baseline creatinine demonstrated a significantly increased hazard ratio (HR) of infection during the year following discharge compared to controls: HR 2.90; 95% CI, 2.10–4.02, p <0.0001 (Fig 3).

## Discussion

This study demonstrates that among patients from an integrated health care delivery system, non-infectious AKI with return to baseline creatinine was associated with increased odds of infection in the year after discharge compared to propensity-score matched controls without AKI. While previous studies have established AKI as a risk factor for subsequent in-hospital infection, little is known about infection rates following discharge. Our results demonstrate that short-term 30 day infection rates are significantly higher among patients with AKI during hospitalization, even though serum creatinine values returned to baseline. Strikingly, while there was no difference in the odds of infection among cases compared to controls at 90 days post discharge, the odds of infection were significantly higher among cases in the period of 91–365 days following discharge, suggesting a long-term association well after serum creatinine had returned to baseline at discharge.

The odds of incident infection among cases decreased in each of the first 30-day comparison time points such that there was no significant difference in the odds of incident infection among cases and control at 61–90 days. However, the magnitude of the OR during the final period (days 91–365) was much larger than the ORs observed during the first three 30-day observation periods. This may be due to the fact that there were more total infectious episodes during this much longer time period, more AKI patients developed CKD by one year, or there could be another late-term effect that should be further investigated. The hazard ratio is lower after censoring patients at the time of first infection, which suggests that AKI patients are at risk for repeated and/or prolonged infections over the course of the year. However, even after accounting for recurrent or prolonged infection, the hazard ratio for infection among those with AKI followed by return to baseline creatinine was still significantly higher compared to controls without AKI.

Previous studies have demonstrated an increased short-term risk of infection following AKI in hospitalized patients[7, 23–26]. One of the earliest studies examined AKI following contrast administration and found that 45% of non-survivors developed sepsis *after* the development of AKI[7]. Increased rates of infection following AKI were also demonstrated among critically ill patients requiring renal replacement therapy (RRT) [24, 25], as well as among adult patients undergoing open-heart surgery[23]. An analysis of the Program to Improve Care in Acute Renal Disease (PICARD) database found that sepsis occurred in 41% patients with a preceding diagnosis of AKI from a non-infectious cause. Furthermore, these patients had a doubling in mortality compared to AKI patients who did not develop sepsis[26]. Our propensity score matched case control study supports these findings and additionally shows that increased infection rates persist after discharge and are independent of creatinine values.

While these infections were initially thought to be limited to the peri-AKI period, several studies have demonstrated that the association between AKI and infection persists in the long-term as well[8, 12, 33]. A subgroup analysis of infection rates beyond three months of discharge among patients with severe AKI requiring dialysis found that even patients who achieved RRT-independence were at increased risk of developing subsequent sepsis[27]. Another retrospective study found that, when compared to matched controls without AKI, rates of active tuberculosis were significantly increased among individuals with severe AKI who had achieved dialysis-independence[20]. In both of these studies, renal recovery was defined as dialysis-independence, rather than a return to baseline kidney function. Final creatinine values for the recovery group were not reported in either study, but it is likely that CKD was present in a number of these patients. Because patients with CKD are immunocompromised and have high rates of infection[34], and because hemodialysis itself may be an immune modulating event[35], it is unclear from these studies whether the long-term increase in infection was a byproduct of ongoing kidney dysfunction (i.e., CKD). To more precisely address this question, we considered only patients with an episode of AKI that did not require dialysis, followed by return to baseline creatinine defined as a creatinine value within 10% of the baseline level. While CKD rates at one year were still higher in the AKI patients with return to baseline creatinine than in controls, the values were nonetheless far lower than those observed in patients following RRT[14]. Finally, we excluded both cases and controls with a discharge ICD-9 codes for infection so that our analyses were not confounded by the possibility of ongoing, recurrent, or partially treated infection.

Our findings suggest that AKI followed by return to baseline creatinine is a risk factor for subsequent infection and are clinically relevant to AKI survivors, who should be counselled regarding this increased risk of post-discharge infection. Several institutions have recently launched post-AKI clinics aimed at reducing rates of CKD, cardiovascular disease, and overall morbidity and mortality among patients who have survived an in-hospital AKI episode[36]. Screening for infection and educating patients about increased infection risks is another step these clinics can take in safeguarding the long-term health of these patients.

Our study has several strengths, which include the long duration of follow-up and detailed participant clinical data. Importantly, our study design only evaluated AKI cases that did not require dialysis, and in which creatinine returned to within 10% of baseline. This criterion avoids confounding due to infectious complications associated with dialysis, which was a limitation of previous studies[37, 38]. We also excluded patients whose index admission was related to infection. This allowed us to evaluate the residual effects from a single episode of AKI on infection rates without confounding by ongoing severe kidney dysfunction or ongoing, recurrent, or partially treated infection. Propensity-score matching allowed us to account for important confounders that are known to predispose to AKI, thus strengthening our ability to establish the relationship between return to baseline creatinine after AKI and subsequent infection.

This study has several limitations. First, several patient factors that may affect risk of post-discharge infection were unavailable such as nursing home admission, presence of indwelling catheters or ports following discharge, and medications. For instance, use of immunosuppressive medications during or after the index hospitalization could not be assessed within our database. Also, despite careful propensity score analysis, we cannot exclude the possibility of residual confounding and that diseases associated with AKI, such as development of CKD, and other factors, such as illness severity (no illness severity score),might also be associated with increased risk of infection. In this regard, however, we controlled for demographics, prior inpatient visits, all components of the Charlson comorbidity score, systolic blood pressure >140 mmHg, admission day out of 365 days (to account for seasonal variations), baseline creatinine, and index hospitalization LOS (to account for illness severity. In addition, we used ICD-9 codes to identify incident infection, which could affect the validity of our results if information was miscoded or incompletely documented. We excluded hospitalizations with an ICD-9 code for infection thus eliminating sepsis and infection-related AKI, but we were unable to otherwise determine exact etiologies of AKI. Whether the patients had further episodes of AKI or additional hospitalizations following the index hospitalization could not be assessed. Lastly, the population studied was comprised mainly of older Caucasian subjects, making the results difficult to generalize to other age groups and races.

In conclusion, short-term incident infection is associated with increased among patients with AKI followed by return to baseline creatinine compared to propensity-score matched controls without AKI. Our results also suggest that long-term rates of infection may be increased, however, whether this is due to repeat AKI, the development of CKD, or other factors could not be determined in this analysis. Further research to define the mechanism by which AKI may increase infection rates is warranted and may lead to targeted interventions to decrease infection following AKI. Such interventions, combined with dedicated post-AKI care, could significantly reduce the high rates of morbidity and mortality observed in this population.

## Supporting information

S1 Table ICD-9 codes for infection that were considered for the main analysis. (DOCX)

S2 Table Major categories of infection. (DOCX)
