# Supplementary material for: Incident infection following acute kidney injury with recovery to baseline creatinine: A propensity score matched analysis
Source: PLoS One. 2019 Jun 24;14(6):e0217935. doi: 10.1371/journal.pone.0217935 (PMC6590794; doi:10.1371/journal.pone.0217935)
Supplement: S1 Table — (DOCX) [file pone.0217935.s001.docx]

Supplementary Table 1. ICD-9 codes for infection that were considered for the main analysis

| **Bacteremia** | | |
| --- | --- | --- |
| 790.7 |  |  |
| **Endocarditis** | | |
| 421 | 421.1 |  |
| 421.0 | 421.9 |  |
| **Gastrointestinal and hepatobiliary infection** | | |
| 540 | 547.01 | 574.60 |
| 540.0 | 574.1 | 574.61 |
| 540.1 | 574.10 | 574.7 |
| 540.9 | 574.11 | 574.70 |
| 541 | 574.3 | 574.71 |
| 566 | 574.30 | 574.8 |
| 008.45 | 574.31 | 574.80 |
| 569.5 | 574.4 | 574.81 |
| 562.11 | 574.40 | 575.0 |
| 574.0 | 574.41 |  |
| 574.00 | 574.6 |  |
| **Genitourinary tract infections** | | |
| 599.0 | 590.1 | 590.8 |
| 590 | 590.10 | 590.80 |
| 590.0 | 590.11 | 590.81 |
| 590.00 | 590.2 | 590.9 |
| 590.01 | 590.3 |  |
| **Joint or bone infections** | | |
| 711 | 730.0 | 730.32 |
| 711.0 | 730.00 | 730.33 |
| 711.00 | 730.01 | 730.34 |
| 711.01 | 730.02 | 730.35 |
| 711.02 | 730.03 | 730.36 |
| 711.03 | 730.04 | 730.37 |
| 711.04 | 730.05 | 730.38 |
| 711.05 | 730.06 | 730.39 |
| 711.06 | 730.07 | 730.8 |
| 711.07 | 730.08 | 730.81 |
| 711.08 | 730.09 | 730.82 |
| 711.09 | 730.2 | 730.83 |
| 711.9 | 730.20 | 730.84 |
| 711.90 | 730.21 | 730.85 |
| 711.91 | 730.22 | 730.86 |
| 711.92 | 730.23 | 730.87 |
| 711.93 | 730.24 | 730.88 |
| 711.94 | 730.25 | 730.89 |
| 711.95 | 730.26 | 730.9 |
| 711.96 | 730.27 | 730.91 |
| 711.97 | 730.28 | 730.92 |
| 711.98 | 730.29 | 730.93 |
| 711.99 | 730.3 | 730.94 |
| 730 | 730.31 | 730.95 |
| 730.96 | 730.98 |  |
| 730.97 | 730.99 |  |
| **Pulmonary infections** | | |
| 480 | 482.32 | 483.0 |
| 480.0 | 482.39 | 483.1 |
| 480.1 | 482.4 | 483.8 |
| 480.2 | 482.40 | 484 |
| 480.3 | 482.41 | 484.0 |
| 480.8 | 482.42 | 484.1 |
| 480.9 | 482.49 | 484.3 |
| 481 | 482.8 | 484.5 |
| 482 | 482.81 | 484.6 |
| 482.0 | 482.82 | 484.7 |
| 482.1 | 482.83 | 484.8 |
| 482.2 | 482.84 | 485 |
| 482.3 | 482.89 | 486 |
| 482.30 | 482.9 | 487.0 |
| 482.31 | 483 |  |
| **Septicemia** | | |
| 038 | 038.19 | 038.42 |
| 038.0 | 038.2 | 038.43 |
| 038.1 | 038.3 | 038.44 |
| 038.10 | 038.4 | 038.49 |
| 038.11 | 038.40 | 038.8 |
| 038.12 | 038.41 | 038.9 |
| **Soft tissue infections** | | |
| 681 | 682 | 682.6 |
| 681.0 | 682.0 | 682.7 |
| 681.00 | 682.1 | 682.8 |
| 681.01 | 682.2 | 682.9 |
| 681.1 | 682.3 | 728.86 |
| 681.10 | 682.4 | 040.0 |
| 681.9 | 682.5 |  |
