# Supplementary material for: Incident infection following acute kidney injury with recovery to baseline creatinine: A propensity score matched analysis
Source: PLoS One. 2019 Jun 24;14(6):e0217935. doi: 10.1371/journal.pone.0217935 (PMC6590794; doi:10.1371/journal.pone.0217935)
Supplement: S2 Table — (DOCX) [file pone.0217935.s002.docx]

Supplementary Table 2. Major categories of infection

| **ICD-9 Code** | **Infection** | **Infection Category** |
| --- | --- | --- |
| 008.45 | C. difficile enteritis | Gastrointestinal and genitourinary |
| 540.0 | Acute appendicitis NOS |  |
| 541 | Appendicitis NOS |  |
| 562.11 | Diverticulitis of colon |  |
| 566 | Anal rectal abscess |  |
| 569.5 | Intestinal abscess |  |
| 574.00 | Cholelithiasis with acute cholecystitis |  |
| 574.10 | Cholelithiasis with cholecystitis NEC |  |
| 575.0 | Acute cholecystitis |  |
| 590.10 | Acute pyelonephritis NOS |  |
| 590.80 | Pyelonephritis NOS |  |
| 599.0 | Urinary Tract Infection NOS |  |
| 038.0 | Septicemia | Septicemia and bacteremia |
| 038.10 | Staph septicemia NOS |  |
| 038.11 | Staph aureus septicemia |  |
| 038.12 | MRSA septicemia |  |
| 038.19 | Other staph septicemia |  |
| 038.3 | Anaerobic septicemia |  |
| 038.40 | Gram-negative septicemia NOS |  |
| 038.42 | E. coli septicemia |  |
| 038.43 | Pseudomonas septicemia |  |
| 038.49 | Gram-negative septicemia NEC |  |
| 038.9 | Septicemia NOS |  |
| 790.7 | Bacteremia |  |
| 040.0 | Gas gangrene | Skin, bone, and joint |
| 681.10 | Cellulitis, toe NOS |  |
| 681.9 | Cellulitis of digit NOS |  |
| 682.0 | Cellulitis of face |  |
| 682.1 | Cellulitis of neck |  |
| 682.2 | Cellulitis of trunk |  |
| 682.3 | Cellulitis of arm |  |
| 682.4 | Cellulitis of hand |  |
| 682.5 | Cellulitis of buttock |  |
| 682.6 | Cellulitis of leg |  |
| 682.7 | Cellulitis of foot |  |
| 682.8 | Cellulitis, site NEC |  |
| 682.9 | Cellulitis NOS |  |
| 711.00 | Pyogenic arthritis, unspecified |  |
| 711.01 | Pyogenic arthritis, shoulder |  |
| 711.91 | Infectious arthritis NOS, shoulder |  |
| 711.96 | Infections arthritis NOS, leg |  |
| 711.97 | Infectious arthritis NOS, ankle |  |
| 728.86 | Necrotizing fasciitis |  |
| 730.07 | Acute osteomyelitis, ankle |  |
| 730.20 | Osteomyelitis NOS, unspecified |  |
| 730.24 | Osteomyelitis NOS, hand |  |
| 730.27 | Osteomyelitis NOS, ankle |  |
| 730.28 | Osteomyelitis NOS, other site |  |
| 730.29 | Osteomyelitis NOS, multiple sites |  |
| 421.0 | Acute/subacute bacterial endocarditis | Endocarditis |
| 421.9 | Acute/subacute endocarditis NOS |  |
| 480.9 | Viral pneumonia NOS | Pneumonia and respiratory tract |
| 481 | Pneumococcal pneumonia |  |
| 482.2 | H. influenza pneumonia |  |
| 482.41 | Staph aureus pneumonia |  |
| 482.82 | Pneumonia, e. coli |  |
| 482.89 | Pneumonia, other specific bacteria |  |
| 482.9 | Bacterial pneumonia NOS |  |
| 484.0 |  |  |
| 485 | Bronchopneumonia, organism NOS |  |
| 486 | Pneumonia, organism NOS |  |
| 487.0 | Influenza with pneumonia |  |
